# Co-infections with *Plasmodium knowlesi* and Other Malaria Parasites, Myanmar

**DOI:** 10.3201/eid1609.100339

**Published:** 2010-09

**Authors:** Ning Jiang, Qiaocheng Chang, Xiaodong Sun, Huijun Lu, Jigang Yin, Zaixing Zhang, Mats Wahlgren, Qijun Chen

**Affiliations:** Author affiliations: Jilin University, Changchun, People’s Republic of China (N. Jiang, Q. Chang, H. Lu, J. Yin, Q. Chen);; Chinese Academy of Medical Sciences, Beijing, People’s Republic of China (X. Sun, Z. Zhang, Q. Chen);; Institute for Parasitic Disease Control, Puer City, People’s Republic of China (X. Sun, Z. Zhang);; Karolinska Institutet, Stockholm, Sweden (M. Wahlgren, Q. Chen)

**Keywords:** Plasmodium knowlesi, malaria, co-infection, Myanmar, parasites, vector-borne infections, dispatch

## Abstract

To determine the frequency of co-infections with *Plasmodium* species in southern Myanmar, we investigated the prevalence of *P. knowlesi*. More than 20% of patients with malaria had *P. knowlesi* infection, which occurred predominantly as a co-infection with either *P. falciparum* or *P. vivax*.

*Plasmodium* species are co-endemic to regions of Southeast Asia (*1**,**2*). This finding is believed to be underestimated because of insufficient sensitivity of microscopic detection of parasites. The prevalence of mixed infections with malaria parasites in the border regions between Thailand and Myanmar was recently found to be <24% (*3*). Identification of *P. knowlesi* as the fifth human malaria pathogen, which is prevalent in countries in Southeast Asia, has complicated this situation. *P. knowlesi* is a parasite that infects mainly long-tailed macaques (*Macaca fascicularis*) and pig-tailed macaques (*M. nemestrina*) in Southeast Asia (*4*). The parasite has developed the capacity to naturally infect humans, and infections in some persons have been life-threatening (*5**,**6*). Furthermore, infections with *P.* *knowlesi* in travelers to this region have been increasing (*7**,**8*).

*P. knowlesi* isolates obtained from humans have been frequently misidentified as *P. falciparum* or *P. malariae* because of the morphologic similarities of these parasites (*2*). Use of PCRs specific for 18S small subunit (SSU) rRNA genes of malaria parasites has identified suspected cases (*2**,**9*). *P. knowlesi* infection in humans in the border area between the People’s Republic of China and Myanmar has been reported (*10*), but the prevalence is unknown. We investigated the frequency of co-infections with *P. knowlesi* and other *Plasmodium* spp. in this region.

## The Study

The study was reviewed and approved by the Ethic Committee of the Institute for Parasitic Disease Control of Yunnan Province and local administration authority in Myanmar. One hundred forty-six blood samples were obtained in 2008 from randomly selected patients with uncomplicated malaria in southern Myanmar near Yunnan Province of China, where pig-tailed macaques are also present. Written consent was obtained from each person before blood samples were obtained. A drop (20 µL–50 µL) of fingerprick blood was placed directly on premarked filter paper. Malaria infection was identified by microscopic analysis of Giemsa-stained blood films made from blood spotted on the paper.

DNA templates for a nested PCR were prepared from whole blood spots on filter paper according to a previously reported method (*11*). Genomic DNA of *P. falciparum* was obtained from in vitro–proliferated 3D7 clone. Genomic DNA of *P. vivax* was obtained from a patient from the study region previously identified by using PCR and the primer sets used in this study. As reported (*2**,**12*), DNA (3 μL/sample and 1 ng of each positive control DNA) from each sample was amplified with the *Plasmodium* genus–specific primer pair rPLU1 and rPLU5.

Two microliters of PCR product from each amplification was subjected to a second PCR amplification with species-specific primer pairs rFAL1 and rFAL2 for *P. falciparum*, rMAL1 and rMAL2 for *P. malariae*, rVIV1 and rVIV2 for *P. vivax*, rOVA1 and rOVA2 for *P. ovale*, and Pmk8 and Pmkr9 for *P. knowlesi*. PCR products amplified with nested primers were analyzed by agarose gel electrophoresis. DNA bands were removed from the gel, purified by using the QIAquick Gel Extraction Kit (QIAGEN, Valencia, CA, USA) and ligated to T-cloning vector (Invitrogen, Carlsbad, CA, USA) according to protocols provided by the manufacturers. Plasmid inserts were then sequenced.

Sequence identity was confirmed by random basic local alignment search tool analysis of sequences in GenBank (http://blast.ncbi.nlm.nih.gov/). Novel sequences were deposited in GenBank with accession nos. GU816242–GU816250. Phylogenetic relationships of unique sequences amplified by using nested primers with corresponding reference sequences were constructed by using the neighbor-joining method in MEGA version 4.0 (*13*). All sequences clustered with reference sequences of *P. falciparum*, *P. vivax*, or *P. knowlesi*, which suggested that all sequences were species specific (Figure).

**Figure:**
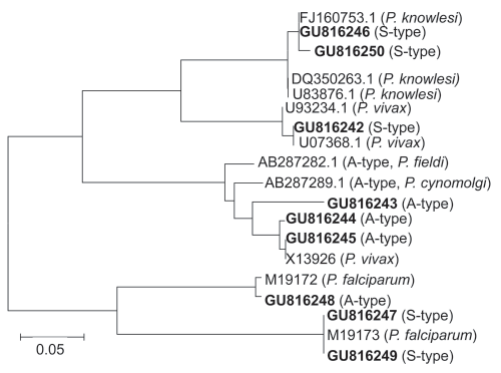
Phylogenetic analysis of A-type and S-type 18S small subunit (SSU) rRNA gene sequences of *Plasmodium* spp., Myanmar, 2008. Fragments of 18S SSU rRNA gene sequences of samples were analyzed by aligning with published homologous sequences of *P. falciparum*, *P. vivax,* and *P. knowlesi.* A phylogenetic tree was constructed on the basis of similarities by using MEGA version 4.1 (*13*). Novel sequences identified in this study are indicated in **boldface**. Scale bar indicates nucleotide substitutions per site.

## Conclusions

Three parasite species (*P. falciparum*, *P. vivax*, and *P. knowlesi*) were identified in 146 infected persons. Phylogenetic analysis showed that amplified products were species specific (Figure). Monoinfection with *P. falciparum*, *P. vivax*, and *P. knowlesi* accounted for 34.9% (51/146), 36.3% (53/146), and 2.7% (4/146), respectively, of the infections. Mixed infections of *P. knowlesi* with *P. falciparum* or *P. vivax* accounted for 6.9% (10/146) of the infections, and mixed infections with *P. knowlesi* and either *P. falciparum* or *P. vivax* accounted for 8.9% (n = 13 in both groups) of the infections. Only 2 samples (1.4%) had mixed infections with *P. falciparum*, *P. vivax*, and *P. knowlesi* (Table). Thus, the prevalence of mixed infections in southern Myanmar was lower than that in northern Myanmar near the border with Thailand (*3*). The prevalence of *P. knowlesi* was 21.9%. In most cases, this parasite showed co-infection with either *P. falciparum* or *P. vivax*, which indicated that *P. knowlesi* may have not fully adapted to the human host or that humans who were infected with other malaria parasites may be more vulnerable to *P. knowlesi* infection.

**Table:** *Plasmodium* species identified in 146 persons, Myanmar, 2008

Parasite	No. (%) persons
*Plasmodium knowlesi*	4 (2.7)
*P. knowlesi*/*P. falciparum*	13 (8.9)
*P. knowlesi*/*P. vivax*	13(8.9)
*P. knowlesi*/*P. falciparum*/*P. vivax*	2(1.4)
*P. falciparum*	51 (34.9)
*P. vivax*	53 (36.3)
*P. falciparum*/*P. vivax*	10 (6.9)

Our study also emphasizes the need for improvement of current methods for detecting *P. knowlesi* infection (*14**,**15*). A recent report found that the primer pair Pmk8 and Pmkr9, which is specific for the 18S SSU rRNA gene of *P. knowlesi*, can cross-hybridize with the corresponding sequence of *P. vivax* (*14*). We observed weak amplifications in 16 samples (11%); all were from *P. vivax*–infected blood. All amplicons made by using Pmk8 and Pmkr9 primers were sequenced and compared with the homologous sequences. Because of the high similarity of 18S SSU rRNA gene sequences among these parasites, more specific sequences are needed for establishing a reliable PCR-based method for routine diagnosis of *P. knowlesi* infection (*14*).
